# ThinPrep cytology combined with HPV detection in the diagnosis of cervical lesions in 1622 patients

**DOI:** 10.1371/journal.pone.0260915

**Published:** 2021-12-02

**Authors:** Sulaiya Husaiyin, Zhen Jiao, Kailibinuer Yimamu, Reyilanmu Maisaidi, Lili Han, Mayinuer Niyazi

**Affiliations:** Department of Gynecology, People’s Hospital of Xinjiang Uygur Autonomous Region, Urumqi, China; Ruđer Bošković Institute, CROATIA

## Abstract

The timely detection of precancerous lesions and early intervention can greatly reduce cervical cancer occurrence. The current study aimed to assess the diagnostic value and accuracy of different methods of cervical lesion screening. A total of 1622 females who visited the Outpatient Department of Xinjiang Uyghur Autonomous Region People’s Hospital between January and December 2018 were consecutively enrolled. All participants underwent separate high-risk human papilloma virus (HR-HPV) DNA detection, ThinPrep cytology testing (TCT) and colposcopic biopsy. Their medical records were retrospectively analyzed. While considering biopsy outcomes as the gold standard, the diagnostic values of TCT, HR-HPV testing, and TCT+HR-HPV testing for cervical cancer screening were compared. The sensitivity, specificity and Youden index of each method were calculated. Among the different methods, TCT+HR-HPV testing had the highest sensitivity (89.8%), followed by TCT (79.9%) and HR-HPV testing (49.2%). The combined method also had the highest Youden value, and its screening outcomes exhibited the highest consistency with those of biopsy. In addition, the combined method had the largest area under the receiver operating characteristic (ROC) curve, which was 0.673 (0.647, 0.699), compared with any other screening method. Compared with TCT or HR-HPV testing alone, TCT+HR-HPV testing serves as a better screening method for cervical cancer and precancerous lesions.

## Introduction

Cervical cancer is the fourth most common malignancy in terms of incidence and mortality in women worldwide. In 2018, a total of 570,000 new cases of cervical cancer were diagnosed worldwide, and the number of deaths due to cervical cancer reached 311,000; among cervical cancer-related deaths, approximately 85% occurred in developing and undeveloped countries, and the mortality was 18 times higher in low–middle-income countries than high-income countries [1].

China is the largest developing country in the world, and cervical cancer is highly prevalent in China. Annually, approximately 130,000 new cases of cervical cancer are diagnosed in China, which accounts for 28.8% of all new cases worldwide; the annual number of cancer-related deaths in China is approximately 80,000, and the mortality ranks second only to that caused by breast cancer [2, 3]. Xinjiang is a region of China with a high prevalence of cervical cancer, particularly among Uyghur women. The incidence of cervical cancer among Uyghur women is approximately 527/100,000, which is significantly higher than the average incidence nationwide [4]. In Xinjiang, cervical cancer is the leading cause of death among various female cancers and poses a serious threat to the health and life expectancy of women. According to the literature, the human papillomavirus (HPV) detection rate of cervical samples from Uyghur females is high, particularly that of HPV-16 [5, 6]. The development of cervical cancer can be affected by a variety of factors, such as socioeconomics and ethnicity [7]. Xinjiang is a multiethnic autonomous region in China. The population and geographic environment of different ethnic groups and regions are varied. The prevalence of cervical cancer in different regions of Xinjiang, such as southern Xinjiang, eastern Xinjiang and northern Xinjiang, also differs [8, 9].

The progression of cervical cancer from cervical intraepithelial neoplasia (CIN) is a long, reversible process, and this period is referred to as the precancerous stage. If disease is detected at this stage (i.e., early) and timely intervention is then provided, the progression of CIN may be prevented, thereby reducing the possibility of cervical cancer occurrence [6].

HPV infection is the primary cause of precancerous lesions and cervical cancer; high-risk HPV (HR-HPV) infection is the main risk factor for high-grade CIN and cervical cancer, which has been evidenced by research on both pathogenesis and vaccines [4, 10, 11]. To date, more than 100 types of HPV have been identified and are classified as high-risk and low-risk types. Among these HPVs, more than 10 high-risk types have been identified to be closely associated with the development of cervical cancer and precancerous lesions; particularly among sexually active women, the virus infection rate is high. In most individuals, HPV spontaneously is spontaneously resolved by the host immune system; however, in some cases, the virus persists and eventually leads to cancer [12, 13]. As early as 1999, the U.S. Food and Drug Administration (FDA) approved HPV DNA testing as an auxiliary examination method for atypical squamous cells of undetermined significance (ASC-US); since then, the related understanding of HPV DNA testing has been continuously updated and optimized [14–16]. HR-HPV testing has also been applied in cervical cancer in recent years; however, for most developing countries, this technique has not been popularized as the primary screening test in clinical practice [17].

In this study, we compared the screening value and accuracy of ThinPrep cytology testing (TCT), HR-HPV DNA testing and TCT combined with HR-HPV testing (TCT+HR-HPV) in cervical cancer screening for women in Xinjiang. The results of this study may provide a fast, accurate cervical cancer screening method for the population of Xijiang, as well as for those in other regions with similar socioeconomic backgrounds.

## Materials and methods

### Study subjects

**Fig 1:**
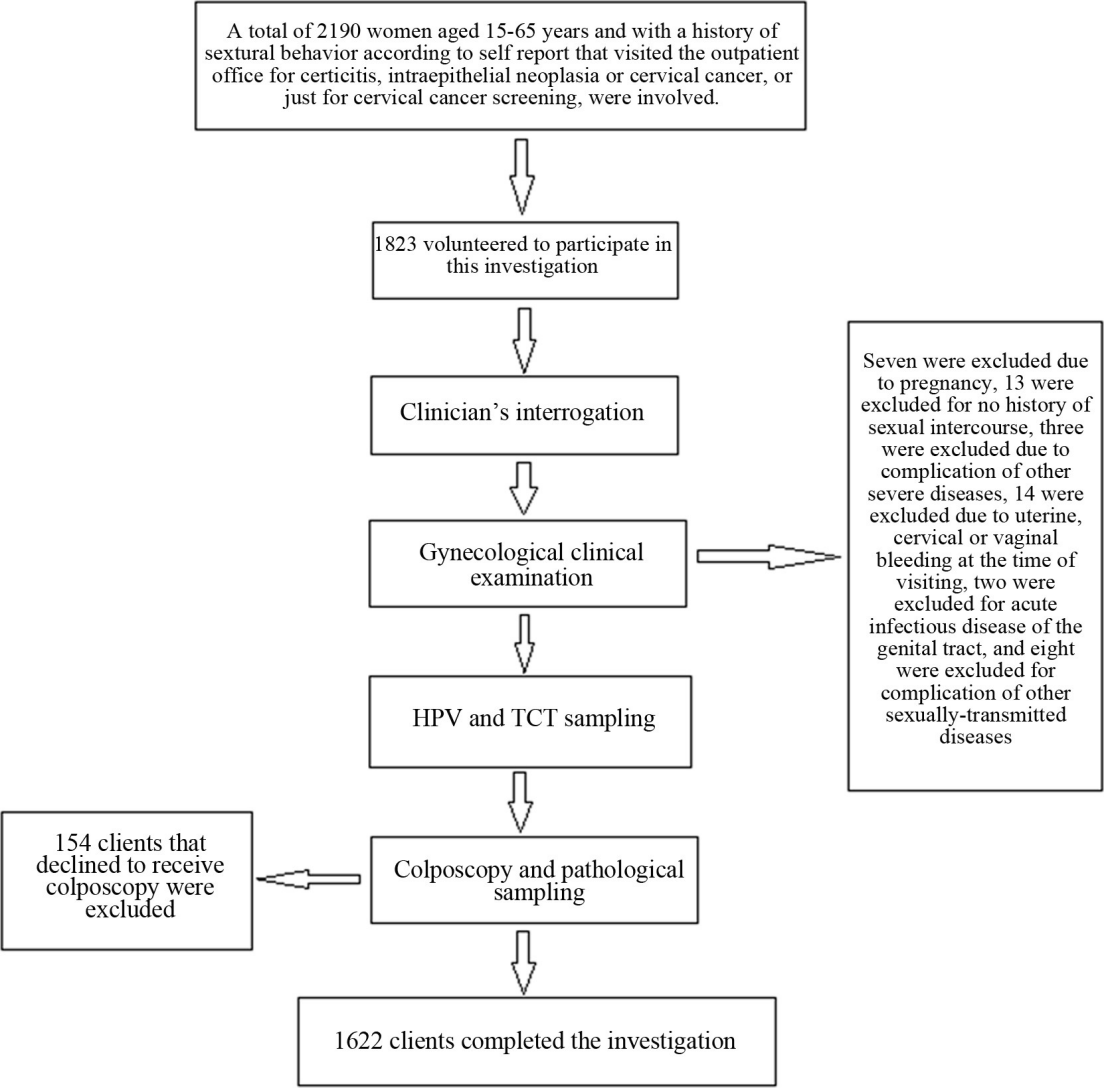
Flow chart of the recruitment for this study.

Between January and December 2018, a total of 2190 women who visited the outpatient office of the Department of Gynecology of Xinjiang Uyghur Autonomous Region People’s Hospital for cervicitis, intraepithelial neoplasia or cervical cancer or only for cervical cancer screening were considered for inclusion in this study. They were informed of the research goals and voluntarily completed a series of separate examinations, including HR-HPV testing, TCT and pathological biopsy. Their ages ranged from 15 to 65 years old. All participants met the following inclusion criteria: 1) aged between 18 years and 65 years; and 2) a history of sexual intercourse (based on inquiring whether the patient had such a history as well as observing the hymen condition at the time of sample collection). The exclusion criteria were as follows: 1) pregnancy or pregnancy termination within 3 months prior to this study; 2) refusal to receive colposcopic biopsy or dysplasia of the cervix according to colposcopic biopsy at the time of the current visit; 3) no history of sexual intercourse; 4) mental disorders or other severe diseases; 4) uterine, cervical or vaginal hemorrhage at the time of diagnosis; 5) acute lower genital tract, vulvar, vaginal or cervical infection; and 6) other concurrent sexually transmitted diseases. Finally, 1622 females qualified for this study. Their medical records included general and clinical data and were retrospectively analyzed between December 1, 2019, and December 31, 2019. A flow chart describing recruitment is shown in Fig 1.

The procedures of this study were approved by the Ethics Committee of Xinjiang Uyghur Municipal People’s Hospital (KY20180118164). Written informed consent for research purposes was obtained from each participant.

### Laboratory tests

#### ThinPrep cytology testing (TCT)

Exfoliated cells at the external aperture of the cervix and the cervical canal were collected with a sampling brush and then stored in a bottle with preservation solution. A thin-layer cell smear was made with a ThinPrep 2000 system for Pap examinations. Diagnoses were made according to the grading criteria of the Bethesda System: 1) no intraepithelial or malignant lesions (NILM); 2) squamous epithelial cell abnormality: a, atypical squamous cells (ASCs), including ASC-US and atypical squamous cells that cannot exclude high-grade squamous intraepithelial lesion (ASC-H); b, low-grade squamous intraepithelial lesion (LSIL); c, high-grade squamous intraepithelial lesion (HSIL); and d, squamous cell carcinoma (SCC); 3) glandular epithelial cell abnormality: a, atypical glandular cells (AGCs), including AGC-not otherwise specified (AGC-NOS) and AGC-suspicious for neoplasia (AGC-N); b, cervical adenocarcinoma in situ of the cervical canal (AIS); and c, adenocarcinoma; and 4) other malignant tumors.

#### High-risk human papilloma virus (HR-HPV) testing

Viral DNA was amplified using real-time fluorescent quantitative polymerase chain reaction (RT-PCR), and the procedures were performed with the HPV genotyping kit with specificity of 98%, accuracy of 98% and repeatability of 98% (Yaneng Biosciences (Shenzhen) Co., Ltd., China). The universal primers for fragment amplification of HPV L1 genes were 5’-CGTCCMARRGGAWACTGATC-3’ (MY09) and 5’-GCMCAGGGWCATAAYAATGG-3’ (MY11), and the primers for *β*-globin amplification were 5’-ACA CAA CTG TGT TCA CTA GC-3’ (F) and 5’-CAA CTT CAT CCA CGT TCA CC-3’ (R). Viral genes and types were determined using reverse dot-blot (RDB) hybridization (this technique can identify 15 HR-HPV types, i.e., 16, 18, 31, 33, 35, 39, 45, 51, 52, 53, 56, 58, 59, 66, and 68). According to the criteria indicated in the kit, HR-HPV >1.0 pg/ml was considered positive.

#### Colposcopy and pathological biopsy

Colposcopy was performed after coating with 3% acetic acid. Iodine staining was performed. For each participant, four tissue samples were taken from the iodine-uncolored regions for histopathological examination (specifically, at 3, 6, 9 and 12 o’clock of the normal transformation zone). For those with suspected lesions, based on the outcomes of acetic acid coating and iodine staining, extra tissue was taken from the abnormal site. The pathological diagnosis criteria for CIN and carcinoma in situ were as follows: 1) CIN-I, mild dysplasia; 2) CIN-II, moderate dysplasia; 3) CIN-III, severe dysplasia, including carcinoma in situ; 4) carcinoma in situ and early infiltration; and 5) infiltrating carcinoma, including squamous carcinoma and adenocarcinoma.

#### Outcome determination

The judgment criteria for the results of different examination methods were as follows:

1. TCT: all results were positive except for NILM results;
2. TCT+HR-HPV: if the result of either method was positive, the final result was considered positive; a final result was considered negative only when the results of both methods were negative.

If a patient was diagnosed with ASC-US or LSIL according to TCT and with LSIL or other benign lesions according to pathological biopsy, the TCT outcome was considered “consistent”. If the TCT outcome was the same but the patient was diagnosed as HSIL or a condition more severe than HSIL, the TCT outcome was considered an “insufficient diagnosis”. If the TCT outcome for a patient was ASC-H, HSIL or SCC and that of pathological biopsy was HSIL or SCC, the TCT outcome was considered “consistent”. However, if the TCT outcome remained the same but the pathological biopsy outcome was LSIL or a condition less severe than LSIL, the TCT outcome was considered an “overdiagnosis”. Both “insufficient diagnosis” and “overdiagnosis” were considered an “inconsistent diagnosis”.

### Statistical analysis

Data were processed with SPSS 21.0. While considering the outcomes of colposcopic biopsy to be the gold standard, the diagnostic values of TCT, HR-HPV DNA testing and TCT+HR-HPV testing were compared with the chi-square test. The sensitivity, specificity, Youden index and consistency of each approach were determined. A difference of P<0.05 was considered statistically significant.

## Results

### Baseline characteristics of the subjects

A total of 1622 females received simultaneous TCT, HR-HPV testing and colposcopic biopsy. Their baseline information is summarized in Table 1. Their age was 36.9±9.5 years. Among these individuals, 864 were Han, 547 were Uyghur, 124 were Hazakh, and 87 were other nationalities. HR-HPV positivity was detected in 680 patients, which represented 41.9% of all participants. According to TCT, 1124 patients (69.3% of patients) had ASC-US or a condition more severe than ASC-US. According to pathological biopsy, 762 patients had CIN-I or above, and 24 of these patients had cervical cancer (47.0% and 1.48% of all patients, respectively).

**Table 1:** Baseline information of the subjects.

Variable	Number of patients (%)	Variable	Number of patients (%)
**Nationality**		**Education level**	
Han	864 (53.3%)	Primary school or below	230 (14.2%)
Uyghur	547 (33.7%)	Middle school	531 (32.7%)
Hazakh	124 (7.6%)	College	362 (22.3%)
Other nationalities	87 (5.4%)	University or above	499 (30.8%)
**Number of pregnancies**		**Occupation**	
0	188 (11.6%)	Professional woman	825 (50.9%)
1–3	880 (54.3%)	Housewife	446 (27.5%)
4–6	501 (30.9%)	Farmer	189 (11.7%)
6 or more	53 (3.3%)	Retired	148 (9.1%)
**Number of births**		Student	14 (0.9%)
0	255 (13.9%)	**Location**	
1–2	1121 (69.1%)	Urumqi	976 (60.2%)
3–4	206 (12.7%)	Non-Urumqi	646 (39.9%)
5 or more	40 (2.5%)		
**Chief complaint**			
Health examination	556 (34.3%)		
Abnormal leukorrhea or vaginal bleeding	138 (8.5%)		
Cervical lesions or space-occupying lesions	535 (33.0%)		
Others	393 (24.2%)		

### TCT performance

The detection rates of TCT for CIN-I, CIN-II, CIN-III, and cervical cancer were 64.7% (154/238), 88.3% (309/350), 81.3% (122/150), and 100.00% (24/24), respectively (Table 2; S1 File).

**Table 2:** Analysis of the TCT outcomes and pathological examination outcomes for 1622 patients with cervical lesions.

TCT outcome	Case number (%)	Colposcopy and pathological examination outcomes				
		Normal or inflammatory	CIN-I	CIN-II	CIN-III	Cervical cancer
NILM	498 (100%)	345 (69.3%)	84 (16.9%)	41 (8.2%)	28 (5.6%)	0 (0.0%)
ASC-US	561 (100%)	358 (63.8%)	88 (15.7%)	69 (12.3%)	46 (8.2%)	0 (0.0%)
ASC-H	173 (100%)	49 (28.3%)	13 (7.5%)	89 (51.4%)	22 (12.7%)	0 (0.0%)
LSIL	217 (100%)	89 (41.0%)	44 (20.3%)	55 (25.3%)	29 (13.4%)	0 (0.0%)
HSIL	134 (100%)	15 (11.2%)	4 (3.0%)	82 (61.2%)	18 (13.4%)	15 (11.2%)
SCC	39 (100%)	4 (10.3%)	5 (12.8%)	14 (35.9%)	7 (17.9%)	9 (23.1%)
Total	1622 (100%)	860 (53.0%)	238 (14.7%)	350 (21.6%)	150 (9.2%)	24 (1.5%)

Notes: NILM, no intraepithelial or malignant lesions; ASC-US, atypical squamous cells of undetermined significance; ASC-H, atypical squamous cells that cannot exclude high-grade squamous intraepithelial lesion; LSIL, low-grade squamous intraepithelial lesion; HSIL, high-grade SIL; SCC, squamous cell carcinoma.

### HPV testing performance

The detection rates of HPV testing for CIN-I, CIN-II, CIN-III, and cervical cancer were 38.2% (91/238), 50.3% (176/350), 59.3% (89/150), and 79.2% (19/24), respectively (Table 3; S1 File).

**Table 3:** Analysis of HPV testing outcomes and pathological examination outcomes for 1622 patients with cervical lesions.

HPV outcome	Case number (%)	Colposcopy and pathological examination outcomes				
		Normal or inflammatory	CIN-I	CIN-II	CIN-III	Cervical cancer
Negative	942 (100%)	555 (58.9%)	147 (15.6%)	174 (18.5%)	61 (6.5%)	5 (0.5%)
Positive	680 (100%)	305 (44.9%)	91 (13.4%)	176 (25.9%)	89 (13.1%)	19 (2.8%)
Total	1622 (100%)	860 (53.0%)	238 (14.7%)	350 (21.6%)	150 (9.2%)	24 (1.5%)

### TCT+HPV testing performance

The detection rates of TCT+HPV testing for CIN-I, CIN-II, CIN-III, and cervical cancer were 86.5% (206/238), 94.0% (329/350), 83.3% (125/150), and 100% (24/24), respectively (Table 4; S1 File).

**Table 4:** Analysis of the TCT+HPV testing and pathological examination outcomes for 1622 patients with cervical lesions.

TCT+HPV outcome	Case number (%)	Colposcopy and pathological examination outcomes				
		Normal or inflammatory	CIN-I	CIN-II	CIN-III	Cervical cancer
Negative	477 (100%)	399 (83.6%)	32 (6.7%)	21 (4.4%)	25 (5.2%)	0 (0.0%)
Positive	1145 (100%)	461(40.3%)	206 (18.0%)	329 (28.7%)	125 (10.9%)	24 (2.1%)
Total	1622 (100%)	860 (53.0%)	238 (14.7%)	350 (21.6%)	150 (9.2%)	24 (1.5%)

### Comparison of the diagnostic values of different screening strategies

**Fig 2:**
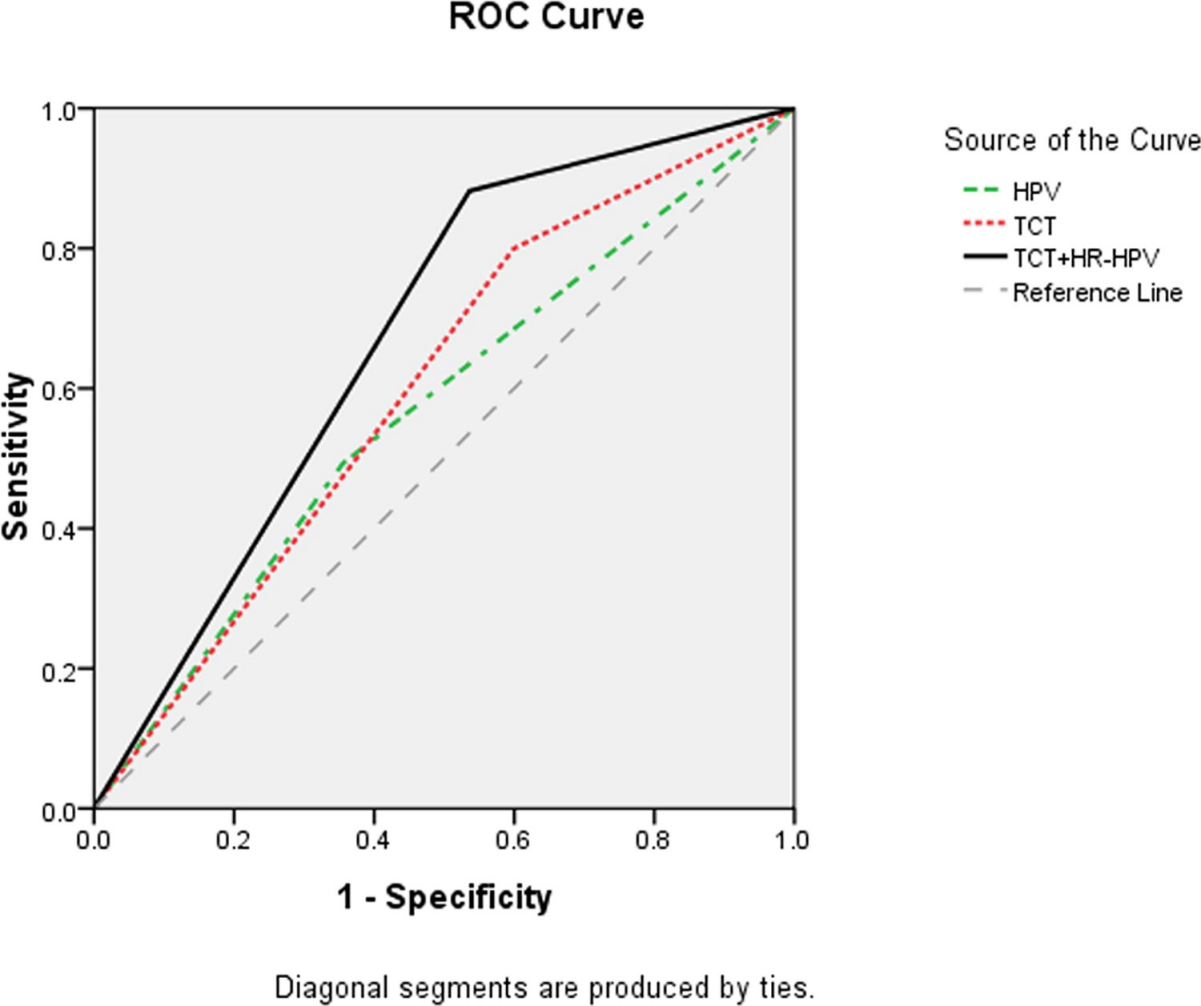
Receiver operating characteristic (ROC) curves of different screening strategies for 1622 patients with cervical lesions.

The diagnostic values of the three screening strategies were then compared. Among them, HR-HPV testing had the highest specificity (64.5%) but the lowest sensitivity (49.2%). In contrast, TCT+HR-HPV testing achieved a sensitivity of 89.8%. Furthermore, the Youden index and consistency of TCT+HR-HPV testing were both higher than those of any other strategy (Table 5). In addition, according to the receiver operating characteristic (ROC) curves, the area under the curve (AUC) of TCT+HR-HPV testing (0.673 (0.647, 0.699)) was the largest (Fig 2, Table 6).

**Table 5:** Analysis of the screening effectiveness of different strategies for 1622 patients with cervical lesions.

		Pathology		Total	Sensitivity	Specificity	Youden index	Consistency
		-	+					
TCT	-	345	153	498	79.9%	40.1%	20.0%	58.8%
	+	515	609	1124				
HPV	-	555	387	942	49.2%	64.5%	13.7%	57.3%
	+	305	375	680				
TCT+HR-HPV	-	399	78	477	89.8%	46.4%	36.2%	66.1%
	+	461	684	1145				
Total		860	762	1622				

**Table 6:** The areas under the curve (AUCs) of different screening strategies for 1622 patients with cervical lesions.

Variable(s)	AUC (95% CI)	Std. Error	Asymptotic Sig
HPV	0.569 (0.541,0.597)	0.014	0.000
TCT	0.600 (0.573,0.628)	0.014	0.000
TCT+HR-HPV	0.673 (0.647,0.699)	0.013	0.000

## Discussion

Although TCT and HPV testing have been widely applied in cervical cancer screening in China, comparisons of the diagnostic value of different screening methods based on a large sample size have rarely been reported. In this study, we compared the diagnostic values of TCT, HR-HPV testing and TCT+HR-HPV testing in a sample size of 1622 patients.

According to this study, the Youden index and consistency level of TCT+HR-HPV testing were higher than those of any other method. In the meantime, the AUC for TCT+HR-HPV testing was also the largest among the three assessed methods. These findings indicate that TCT+HR-HPV testing has the highest outcome accuracy, followed by TCT. In terms of sensitivity, TCT+HR-HPV testing had the most powerful ability to accurately identify cervical cancer, whereas HR-HPV testing had the weakest ability. In addition, our study showed that HR-HPV testing had the highest specificity, whereas TCT had the lowest specificity, which indicates that HR-HPV testing has the most powerful capacity for accurate noncervical cancer screening.

Precancerous lesion detection acts as a critical early warning for the risk of cervical cell cancerization and for the timely prevention and treatment of cervical cancer at an early stage. This study showed that although TCT had a low CIN-I detection rate, its detection rates for CIN-II and above were high (both exceeding 80%), particularly for cervical cancer, which reached 100%. HR-HPV testing had relatively low detection rates for CIN-I through CIN-III, and its detection rate for cervical cancer was lower than 80%. TCT+HR-HPV testing had high detection rates for CIN-I through CIN-III, and its detection rate for cervical cancer reached 100%. These findings indicate that TCT+HR-HPV testing is highly valuable for precancerous lesion screening. Long et al. [18] compared the diagnostic values of TCT, HR-HPV testing, and TCT+HR-HPV testing for cervical cancer among 687 patients and found that the combined method was beneficial for the early screening of cervical cancer. TCT+HR-HPV testing could be a reliable strategy for cervical cancer and CIN screening [19].

Cervical epithelial cells undergo a series of pathological changes before cervical cancer occurs; effective screening during this period can greatly reduce the incidence rate of the malignancy [20]. The WHO has also recommended screening to reduce the incidence of cervical cancer. To date, a number of screening strategies have been proposed, and each strategy has merits and drawbacks. Currently, TCT is the primary cervical cancer screening method and plays an important role in detecting early cervical lesions. According to this study, the sensitivity of TCT was lower than that of the combined screening method, which led to a certain missed diagnosis rate. HPV infection is closely associated with cervical cancer. However, HPV DNA testing can lead to a missed diagnosis rate for cervical cancer of approximately 19%; even after patients are infected with HPV, their HPV testing results may not be positive, as the positivity of HPV testing can be affected by multiple factors, such as the presence of cancer [21]. According to a life-time study among the nonselected Finnish females [22], approximately 50% experience no less than one HPV infection within 10 years, and the females that contract no less than one HPV infection between 20 years and 79 years reach a proportion as high as 79%. For most females, the virus can be cleared by the autoimmune system after infection. Persistent HR-HPV infection, however, can cause cervical cell lesions, which may ultimately lead to cervical cancer. Although colposcopic biopsy serves as the gold standard method for cervical lesions, diagnosis based on this method is complex, and the cost is high. In addition, a colposcopic examination and biopsy can be a rather invasive procedure for patients.

In this study, the HR-HPV positivity rate exceeded 40%, which was noticeably higher than that reported by Wang et al. [23]. In Wang et al.’s study, a total of 3700 participants were included, and the participants included healthy individuals who underwent health examinations as well as patients who received treatment. The rate of our study was also higher than that reported by Pan et al. [24] and by Yao et al. [12]. However, our result was lower than that of samples with abnormal cytological outcomes collected at the Affiliated Hospital of Xinjiang Medical University [25]. In addition, this study showed that the cervical cancer detection rate was 1.48%, which was higher than that reported by Husaiyin et al. (who conducted cervical cancer examinations in Pishan County) [26] and by Han et al. (whose study was conducted in Urumqi) [27]. Different sample sources could account for these inconsistencies. Furthermore, the HPV test itself might also contribute to the differences in the HR-HPV positivity rate between our study and those reported in literature. For instance, in Pan et al.’s study [24], the kit was able to test 23 HPV types, whereas in our study, only 15 types were tested. In addition, some of the subjects in this study already presented with cervical lesions at the time of this investigation, which was another important factor related to the difference in the cervical lesion detection rate compared with studies focusing on lesion screening.

This study had some limitations. First, this was a single-center study. Therefore, to verify the results of this study, multicenter studies need to be conducted in the future. Second, this study focused on the efficacy of different screening methods rather than investigating the prevalence rate of cervical cancer in the involved region. Furthermore, the included individuals were all from our hospital; that is, the samples were not based on the whole population in the involved region. Therefore, the obtained prevalence rates of HPV positivity and cervical cancer in this study may not represent those of the involved region. Third, despite the positive results obtained in this study, laboratory tests only serve as adjuvant measures for clinical diagnosis, and cervical diseases can only be confirmed according to clinical symptoms and pathological examination.

In conclusion, TCT+HR-HPV testing improves the accuracy of cervical lesion screening in clinical practice. Therefore, it can be popularized and integrated as management guidelines in Xinjiang, China, where the incidence of cervical cancer is high. Furthermore, it can be used as an important screening approach for cervical cancer and precancerous lesions.

## Supporting information

S1 File (XLSX)
